# The Hedonic Experience Associated with a Gentle Touch Is Preserved in Women with Fibromyalgia

**DOI:** 10.3390/jcm13185649

**Published:** 2024-09-23

**Authors:** Sofia Tagini, Giorgia Varallo, Paolo Capodaglio, Alessandro Mauro, Federica Scarpina

**Affiliations:** 1 “Rita Levi Montalcini” Department of Neurosciences, University of Turin, Via Cherasco, 15, 10126 Turin, Italy; sofia.tagini@unito.it (S.T.); alessandro.mauro@unito.it (A.M.); 2 I.R.C.C.S. Istituto Auxologico Italiano, U.O. di Neurologia e Neuroriabilitazione, Ospedale San Giuseppe, Via Cadorna, 90, 28824 Piancavallo, VCO, Italy; 3 Department of Biomedical, Metabolic and Neural Sciences, University of Modena and Reggio Emilia, 41125 Modena, Italy; giorgia.varallo@unimore.it; 4 I.R.C.C.S. Istituto Auxologico Italiano, U.O. di Riabilitazione Osteoarticolare, Ospedale San Giuseppe, 28824 Piancavallo, VCO, Italy; p.capodaglio@auxologico.it; 5 Department of Surgical Sciences, Physical and Rehabilitation Medicine, University of Turin, 10126 Turin, Italy

**Keywords:** affective touch, fibromyalgia, chronic pain

## Abstract

**Background/Objectives**: Although manual therapies can be used for pain alleviation in fibromyalgia, there is no clear evidence about the processing of gentle, affective touch in this clinical condition. In fact, persistent painful sensations and psychological factors may impact the hedonic experience of touch. **Methods:** This observational cross-sectional study compared the subjective experience of affective touch between 14 women with fibromyalgia (age range: 35–70; range of years of education: 5–13) and 14 pain-free women (age range: 18–30; range of years of education: 13–19). The participants rated the pleasantness of slow and fast touches delivered by a brush, the experimenter’s hand, and a plastic stick. Tactile stimuli were either imagined or real to disentangle the contribution of top-down and bottom-up sensory components. Additionally, a self-report questionnaire explored the lifetime experiences of affective touch. **Results:** Akin to healthy counterparts, individuals with fibromyalgia rated slow touches delivered by the experimenter’s hand or a brush as more pleasant than fast touches, regardless of whether they were imagined or real. However, the intensity of pain affects only the imagined pleasantness in our participants with fibromyalgia. Furthermore, despite the fibromyalgia patients reporting fewer experiences of affective touch in childhood and adolescence, this evidence was not associated with the experimental outcomes. **Conclusions:** The hedonic experience of affective touch seems preserved in fibromyalgia despite poor intimate bodily contact in youth. We confirmed that bottom-up and top-down factors contribute to the affective touch perception in fibromyalgia: bodily pain may impact even more the expected pleasure than the actual experience. Future investigations may introduce neurophysiological measures of the implicit autonomic responses to affective touch in fibromyalgia. To conclude, although preliminary, our evidence may be in favor of manual therapies for pain relief in fibromyalgia.

## 1. Introduction

Gentle touch may heal chronic pain [1]. Crucially, the C-Tactile (CT) afferent system, devoted to the processing of the motivational and emotional aspects of touch, which are optimally associated with slow (1–10 cm/s) “affective” tactile sensations [2], might also modulate the individual response to nociceptive stimuli [3,4]. However, the therapeutic potential of pleasant touch in the management of chronic pain remains equivocal, with studies supporting an analgesic effect [5] and others finding no significant impact [6]. The experimental heterogeneity may be related to a dissimilar etiology of pain across clinical conditions, influencing the functioning of CT fibers [7]. Also, chronic pain can persist despite no identifiable tissue damage or antecedent [8], such as in fibromyalgia. This syndrome is characterized by widespread pain, heightened sensitivity to painful stimuli (hyperalgesia), and allodynia [9], although there is no evidence of specific physical or neurophysiological abnormalities [10,11]. Physical therapies incorporating gentle touch seem effective in alleviating core symptoms and improve the quality of life [12,13]. However, our understanding of the experience of affective touch in individuals with fibromyalgia remains limited. Previous research has yielded inconclusive findings, with one study suggesting preserved hedonic experiences associated with affective touch in fibromyalgia patients (i.e., as marked by the preference for slow touches over fast touches) despite a generalized lower appreciation for tactile sensations [14].

Our purpose is to provide updated behavioral evidence about the perceived pleasantness of affective touch in women with fibromyalgia, compared to women with no history of persistent pain. As a crucial novelty, we extended the investigation of the pleasantness of affective touch to that of imagined touches. Mental imagery is a form of perceptual processing, which is not triggered by any external stimulus but results in the representation of sensory information [15,16]. Imagery relies on the high-order neural and functional pathways typically involved in the corresponding sensory perception (e.g., visual or auditory circuits) but does not involve afferent systems and sensory receptors. In the context of the tactile domain, Lucas and colleagues [17] adopted functional magnetic resonance imaging (fMRI) to investigate the brain responses to both experienced and imagined gentle touch to the arm (CT-targeted) and palm (non-CT-targeted, myelinated fast-conducting, A-β afferent nerves [18,19]) surfaces, in order to determine whether these processes recruit overlapping or distinct brain networks. They reported a dissociation between the posterior and anterior insula in the experience and imagination of touch. Specifically, the posterior insula showed activation only during the physical experience of touch; instead, the anterior insula was responsive to both experienced and imagined touch, suggesting that this region may play a role in the processing of the affective meaning of the touch.

Investigating the experience of affective touch by mental imagery in fibromyalgia patients may be essential given their typical hypersensitivity to tactile stimuli [20]. By evaluating the individual response to both real and imagined touch, we might associate any altered experience of affective touch in fibromyalgia either to hedonic (top-down) or purely sensory (bottom-up) abnormal processing, as previously performed in other clinical conditions [21,22].

Furthermore, in this study, we measured participants’ self-reported experiences of affective touch across their lifetimes [23]. Affective touch characterizes intimate relationships since infancy, fostering social bonds. On the other hand, limited experiences of affective bodily contact in childhood and adolescence may lead to altered perceptions of affective touch in adulthood, contributing to interpersonal difficulties [23,24]. Notably, individuals with fibromyalgia often report negative life events and unsatisfactory interpersonal experiences [25,26], which may influence their current perception of affective touch. In summary, our study aims to shed further light on the subjective experiences of affective touch in fibromyalgia, within both the experimental setting and real-life interpersonal interactions.

## 2. Materials and Methods

### 2.1. Participants

Right-handed women diagnosed with fibromyalgia [27] by expert rheumatologists were consecutively recruited from September 2021 to June 2022 at the beginning of their rehabilitative treatment in the involved institution. Exclusion criteria were the presence or history of a neurological or a severe psychiatric disorder as well as the evidence of any skin-related condition possibly affecting tactile sensitivity on the forearm (e.g., eczema, scars). Body mass index (BMI) was recorded (weight and height were measured to the nearest 0.1 kg and 0.1 cm, respectively; BMI was expressed as body mass (kg)/height (m^2^)) since the previous evidence points to a possible interplay between obesity (i.e., BMI > 30 kg/m^2^) and symptoms related to fibromyalgia [28]. For each participant, we collected the perceived overall level of pain intensity on a visual analog scale [29]. Moreover, the level of pain referring to (i) the whole body and (ii) the forearm (i.e., which was the body part targeted during the main experiment) was rated immediately before the experiment. Indices of general wellbeing (Psychological General Wellbeing Index–PGWBI [30]), pain intensity (VAS from 0 to 10), kinesiophobia (Tampa Kinesiophobia Scale [31]), and pain catastrophizing (Pain Catastrophizing Scale–PCS [32]) were collected as routine clinical evaluation. Notably, we were not able to report the onset time of the disease in our participants with fibromyalgia because of the nature of the pain-related symptoms. Indeed, most of the time, the participants were not able to recognize a clear onset; instead, symptoms gradually accumulated over time with no single triggering event.

We included data from healthy women as controls from previously published papers relative to the same task [21,22]. Exclusion criteria were diagnosis of fibromyalgia, rheumatic diseases, or chronic pain; neurological or psychiatric disorder, including clinical depression and anxiety disorders; medical treatments in the previous three months.

### 2.2. Tactile Biography Questionnaire

This measure [23] explores the lifespan experiences of affective touch in close relationships. This measure includes 29 self-report items scored on a Likert scale ranging from 1 (e.g., not at all true for me) to 5 (e.g., extremely true) that evaluate four independent components: the frequency of and satisfaction felt with affective touch (i) in childhood/adolescence and (ii) adulthood, (iii) comfort with and (iv) fondness for interpersonal touch in close relationships. Three additional items explore the feelings experienced in bodily affective interactions, the previous negative/unpleasant experiences involving interpersonal touch, and the participant’s preference for giving and/or receiving affective touch. This complementary information is reported as a supplement to the present work (Supplementary Materials S1, Table S1, and Figure S1). The Italian translation of the questionnaire has been previously provided [21,22]. The questionnaire was completed after the affective touch tasks to avoid any biases.

### 2.3. Affective Touch Paradigm

To investigate affective touch, we adopted the same procedure described by Tagini and colleagues [21,22], in which tactile stimuli were delivered by three different tools: (i) a brush, (ii) the female experimenter’s hand, and (iii) a plastic stick, at two different speeds: (i) slow (3 cm/s) speed, which should optimally elicit the CT fibers deputed to the perception of affective touch, and (ii) fast (18 cm/s) speed, as control condition. Each stimulation lasted three seconds; the slow affective touch consisted of one longitudinal and continuous 9 cm stimulation, and the fast non-affective stimulations consisted of six brief consecutive longitudinal 9 cm stimulations [33]. Our procedure included two experimental tasks (i.e., imagery vs. real), during which participants were blindfolded.

In the imagery task, participants were asked to image the tactile stimuli on the dorsal (i.e., outer) surface of their left forearm. Before the imagery procedure, participants saw explanatory videos showing a female hand delivering fast and slow tactile stimulations with the fingers, the brush, and the stick on a paper cylinder with a 9 cm reference line [21,22]. Then, participants were verbally guided by the experimenter in the imagery procedure: “Please, close your eyes and keep them closed until further notice. Each time, I will tell you which of the touch you should imagine; imagine this touch on your left forearm, the same way you saw it in the video until you hear my stop signal. Then, tell me how pleasant this touch might be for you from zero–not pleasant at all–to 100–extremely pleasant”. Each trial (Tool * Speed) was administered once; overall, the imagery task included six trials, randomized across participants.

The real task was always performed after the imagery task. Tactile stimuli were delivered by a female experimenter on the dorsal surface of the participant’s left forearm. Again, participants were asked to keep their eyes closed and report the level of pleasantness (from 0 to 100) of the tactile stimulations they would feel on their forearm; however, they were not told the tactile stimuli would be the same as those previously imagined. Each trial (i.e., Tool * Speed) was repeated three times in a pseudo-randomized order for each participant (i.e., with no consecutive occurrences of the same trial). Overall, the real touch experimental procedure included eighteen trials.

### 2.4. Analyses

Descriptive statistics were preliminary computed, including means, standard deviations, medians, and frequencies. Differences between the two groups in demographical characteristics were explored through independent sample t-tests. Regarding the TBIO questionnaire, ordinal scores were compared between the two groups with non-parametric Mann–Whitney U tests. Two-sided exact *p*-values ≤ 0.05 were considered statistically significant. Relative to the affective touch paradigm, repeated measure analyses of variance were performed separately for each task (i.e., imagery and real), in which Speed (affective and non-affective) and Tool (brush, hand, and stick) were introduced as within-subject factors, while Group (participants with fibromyalgia vs. controls) was a between-subject factor. In case of significant interactions, we estimated marginal means and applied Bonferroni’s correction for multiple comparisons as a post hoc test. Two-sided exact *p*-values ≤ 0.05 were considered statistically significant.

Successively, we verified the relationship between the experience of affective touch in the experimental setting, from a behavioral perspective, and in real life, as reported in the TBIO questionnaire. First, for both the imagery and the real tasks and each tool, we computed the affective touch sensitivity index, which probes participants’ preference for affective over non-affective touch (as expected according to the literature [34,35,36]) when weighted by the overall pleasantness of touch, according to the following formula:

$$\frac{\left(pleasantness\;affective\;touch-pleasantness\;non\;affective\;touch\right)}{\sum [(pleasantness\;affective\;touch-pleasantness\;non\;affective\;touch)/2]}$$

We used a correlational approach computing non-parametric Spearman’s coefficients to verify the possible relationship between the affective touch sensitivity index for the imagined and real touch delivered by the hand or the brush, and the score was reported as the TBIO for the two groups, independently. According to the previous evidence [23,24], we expected a positive association between the amount of affective touch experienced throughout life and the preference for slow/affective touch over fast/non-affective touch. Thus, one-sided exact *p*-values ≤ 0.05 were considered statistically significant. The same approach was used to explore the relationship of the pleasantness of affective touch with the overall pain intensity (0–10) and the level of pain before testing (1–100) relative to the whole body and the forearm in our participants with fibromyalgia. We expected more painful sensations to be associated with lower pleasantness [36,37]. One-sided exact *p*-values ≤ 0.05 were considered statistically significant.

Finally, we performed a correlational analysis through a non-parametric Spearman’s coefficient to verify the possible relationship between the pleasantness of affective touch in the imagined and real touch for each tool (brush, hand, and stick) in the two groups’ performance. With no a priori predictions, two-tailed exact *p*-values ≤ 0.05 were considered statistically significant.

### 2.5. Sample Size Calculation

This analysis was performed considering the main experimental tasks. According to an a priori sample size calculation [38], fourteen participants in each group should guarantee a statistical power of 0.95, with an alfa of 0.05, when performing a repeated measures ANOVA with a within–between interaction, including six repetitions and two groups, assuming a moderate correlation coefficient of 0.5 between the variables and a medium effect size (f = 0.25).

## 3. Results

### 3.1. Participants

Fourteen women diagnosed with fibromyalgia were enrolled (age in years M = 55.79; SD = 8.73; range: 35–70; education in years M = 9.57; SD = 2.77; range = 5–13; BMI M = 39.63; SD = 5.63; range = 32.6–52.08). On average, the participants with fibromyalgia reported an overall level of pain of 4.93/10 (SD = 1.21). Moreover, they reported a whole-body pain intensity of 47.6/100 (SD = 27) and a pain intensity of 17.3/100 (SD = 27.6) relative to the forearm, at the moment of testing. A Wilcoxon signed-rank test for repeated measures indicated that the pain referring to the whole body was significantly higher than the reported pain in the forearm (median difference = 40, W = 55, *p* = 0.01, r_b_ = 1).

Compared with the fourteen healthy participants (age in years M = 24.64; SD = 3.25; range: 18–30; education in years M = 16.64; SD = 1.87; range = 13–19; BMI M = 22.64; SD = 2.42; range = 19.38–28.35) from the previous database [21,22], our sample of women with fibromyalgia was older [t(26) = 12.51; *p* < 0.001; d = 4.73], had a lower level of education [t(26) = 7.93; *p* < 0.001; d = 3], and a higher BMI [t(26) = 10.37; *p* < 0.001; d = 3.92]. According to the clinical records, our participants with fibromyalgia perceived a low level of quality of life (PGWBI total score: M = 38.57/110, SD = 20.83) and wellbeing relative to all the dimensions considered (PGWBI anxiety: M = 8.50/25, SD = 5.27; PGWBI depression: M = 7.29/15, SD = 4.48; PGWBI positivity: M = 6/20, SD = 3.7; PGWBI self-control: M = 6.29/15, SD = 3.89; PGWBI health: M = 5.21/15, SD = 2.69; PGWBI vitality: M = 5.29/20, SD = 3.69). In line with this observation, twelve of the fourteen participants with fibromyalgia reported clinically relevant anxious and/or depressive symptoms, with eight women already under psychopharmacological treatment (i.e., benzodiazepine and/or SNRI/SSRI drugs). The analgesic treatments being taken included opioids and GABA receptor antagonists. Also, our participants with fibromyalgia reported moderate levels of kinesiophobia (TKS score: M = 27.36/52, SD = 11.13) and pain catastrophizing (PCS score: M = 32.7/52, SD = 15.86).

### 3.2. Tactile Biography Questionnaire

Means and standard deviations for the TBIO scores in each group are reported in Table 1. As illustrated, our participants with fibromyalgia reported significantly less experience of affective touch during childhood and adolescence when compared with healthy controls. The adult experience was rated similarly by the two groups. Finally, no differences were observed between the two groups relative to the overall comfort with and fondness for affective touch.

### 3.3. Affective Touch Paradigm

**Figure 1:**
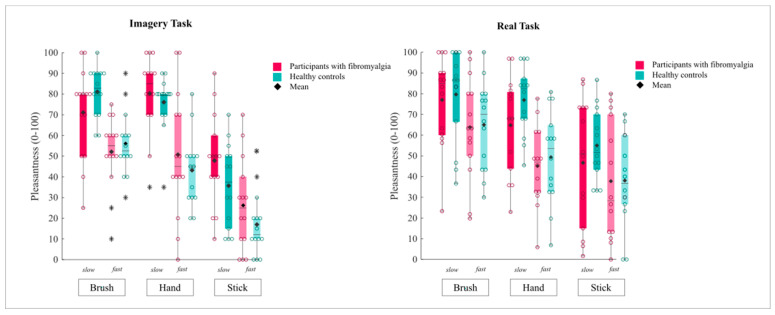
Boxplots illustrate the level of pleasantness experienced (*y*-axis) for affective and non-affective touch in both the imagery (**left**) and real task (**right**), as reported by participants with fibromyalgia (pink) and healthy participants (light blue). Medians are conventionally reported as horizontal lines; diamonds indicate the average pleasantness of affective touch in each experimental condition (Tool * Speed * Gr). Circles indicate the pleasantness reported by each participant in each condition (Tool * Speed * Gr) while asterisks denote outlier values.

Pleasantness ratings in both the imagery and real task are reported in Figure 1.

#### 3.3.1. Imagery Task

Focusing on the experimental within-subject effects, we found a significant main effect of *Tool* [F(2,52) = 59.04; *p* < 0.001; η^2^ = 0.69], indicating that touches delivered by the brush (M = 65.08; SD = 2.55) and the hand (M = 62.58; SD = 2.82) were judged as similarly pleasant [*p* < 0.99], and both were perceived as more pleasant than the touch of the stick (M = 31.74; SD = 3.26) [*p* always >0.001]. Also, we reported a significant main effect of *Speed* [F(1,26) = 52.05 *p* < 0.001; η^2^ = 0.66], since the slow touches (M = 65.35; SD = 2.4) were more pleasant than the fast touches (M = 40.92; SD = 2.99). The interaction Tool * Speed [F(2,52) = 2.28; *p* = 0.11; η^2^ = 0.08] was not significant. Considering the between-subject effect, no significant main effect of *Group* emerged (participants with fibromyalgia M = 51.51; SD = 2.99; healthy controls M = 54.76; SD = 2.99) [F(1,26) = 0.58; *p* = 0.45; η^2^ = 0.22]. Crucially, the first-level interaction Tool * Group [F(2,52) = 3.6; *p* = 0.03; η^2^ = 0.12] was significant. The post hoc comparisons suggested no difference between the groups in rating the pleasantness of each tool [*p* ≥ 0.11]; moreover, in both groups, the pleasantness of the imagined touch delivered by the brush and the hand was similar [participants with fibromyalgia *p* < 0.99; healthy controls *p* = 0.12] whereas all the other comparisons were significant [*p* always <0.001]. The interactions Speed * Group [F(1,26) = 0.1; *p* = 0.74; η^2^ = 0.04] and Tool * Speed * Group [F(2,52) = 0.31; *p* = 0.72; η^2^ = 0.01] were not significant.

#### 3.3.2. Real Task

As regards the experimental within-subject effects, the touches delivered by the brush (M = 71.36; SD = 3.58) were judged as being significantly more pleasant than those of the hand (M = 60.74; SD = 3.04) [*p* > 0.001], and both were more pleasant than the touch of the stick (M = 44.38; SD = 4.38) [*p* > 0.001], as suggested by the significant main effect of *Tool* [F(2,52) = 34.89; *p* < 0.001; η^2^ = 0.57]. Moreover, the significant main effect of *Speed* [F(1,26) = 21.5; *p* < 0.001; η^2^ = 0.45] indicated that the slow touches (M = 67.39; SD = 3.59) were judged as being more pleasant than the fast touches (M = 50.27; SD = 3.79). However, we also observed a significant Tool * Speed interaction [F(2,52) = 3.61; *p* = 0.03; η^2^ = 0.12]. The significant differences observed across the three tools emerged in the case of the slow touches [always *p* ≤ 0.022]; in contrast, fast touches delivered by the hand and the stick were judged as being similarly pleasant [*p* = 0.07], whereas the touch of the brush outperformed that of both tools [always *p* < 0.001]. When we explored the between-subject effects, no significant main effect of *Group* emerged (participants with fibromyalgia M = 61.29; SD = 4.52; controls M = 56.37; SD = 4.52) [F(1,26) = 0.58; *p* = 0.45; η^2^ = 0.22]. Crucially, none of the first-level interactions, Tool * Group [F(2,52) = 0.52; *p* = 0.51; η^2^ = 0.2] and Speed * Group [F(1,26) = 0.62; *p* = 0.43; η^2^ = 0.2], nor the second-level interaction Tool * Speed * Group [F(2,52) = 0.31; *p* = 0.72; η^2^ = 0.01] were significant. 

Overall, these results suggested that our participants with fibromyalgia had a preserved experience of affective touch, in both the real and imagery task.

### 3.4. Correlational Analyses

In Table 2, we report the results of the correlational analyses exploring the relationship between the affective touch sensitivity index and the scores reported in the TBIO questionnaire when considering the touch of the hand and the brush within each group in both the imagined and real task. Our findings suggested no significant relationship between the variables investigated in the group of participants with fibromyalgia or in the healthy controls.

As reported in Table 3, we found a significant relationship between the imagined experience of affective touch and the self-reported level of pain, relative to both the whole body and the forearm in our participants with fibromyalgia; no other significant correlations were observed.

Finally, we investigated the possible relationship between the pleasantness (0–100) of the tactile stimulations in the imagery and the real task for each tool and in each group. A significant positive relationship relative to the pleasantness of the fast touch of the brush in the imagery and the real task was observed in our participants with fibromyalgia (*p* = 0.002, r = 0.57), suggesting that when the brush was tested with the fast touch (i.e., non-affective touch), the imagined tactile sensation may be related to the physical tactile sensation in fibromyalgia. This relationship was not significant in the healthy controls (*p* = 0.24, r = 0.21). No other significant results emerged (all *p*-values > 0.05).

## 4. Discussion

Is the hedonic experience associated with affective touch preserved in fibromyalgia? According to our results, which are grounded on a behavioral approach [21,22], we may offer an affirmative answer. Our participants with fibromyalgia efficiently perceived affective touch: slow touches elicited higher pleasantness than fast touches, as observed in the sample of women with no chronic pain. Our result mirrors the previous evidence reported by Boheme and colleagues [14]. Moreover, it extends this previous evidence to the imaginary experience of affective touch, which was rated as more pleasant than non-affective touch even when imagined. This evidence seems in support of effective touch-based interventions in fibromyalgia: affected individuals may coherently process the hedonic sensations associated with a gentle massage. Furthermore, our participants reported comparable levels of pleasantness when the affective touch was delivered through a brush or by the experimenter’s hand. In other words, both touches were perceived as pleasant. From a clinical perspective, this result suggests that even if a patient would declare discomfort in being touched by the therapist, a tool (such as a soft brush) might actually mediate the hedonic experience of a gentle touch.

A second crucial finding emerged from this research. According to the results registered from the TBIO questionnaire [23], our participants with fibromyalgia reported lower affective touch experiences in childhood and adolescence than the healthy controls did. As illustrated in the Supplementary Materials, our participants with fibromyalgia declared negative or unpleasant experiences of interpersonal touch more frequently than the controls did (Table S1). In fact, relaxing and healing sensations were less commonly associated with affective touch in our experimental sample. In sharp contrast, our participants with fibromyalgia, but not the healthy controls, associated pleasure with interpersonal touch, though both groups generally reported happiness and comfort with interpersonal bodily interactions. Interestingly, embarrassment was the only unpleasant feeling frequently related to affective touch in both groups, whereas rejection, disgust, and annoyance were rarely reported. These results match the evidence of unsatisfying intimate relationships in women with fibromyalgia, especially while growing up. Romeo and colleagues [39] verified that individuals with fibromyalgia described low levels of parental warmth and excessive control during their childhood; both parents were described as negligent, cold, dismissive, and intrusive. Poor emotional connection with both parents, a lack of physical affection, and experiences of parents’ physical quarrels were also reported [40], as well as a high prevalence of maternal abuse and paternal indifference [41]. Then, the adult experience of affective touch seems unaffected in our participants with fibromyalgia, at least when experimentally tested, despite the evidence suggesting that missing or insufficient emotional and bodily closeness might reduce the appreciation of interpersonal touch later in life [23,24]. Furthermore, our participants with fibromyalgia described the amount of affective touch in adulthood similarly to women with no experience of chronic pain. The components of comfort with and fondness for interpersonal touch in close relationships were also similarly rated. The hypothesized relationships between past and present experiences of affective touch might not be linear. In adulthood, individuals can experience satisfying intimacy and closeness with other people despite poor bonding and intimate connections during childhood and adolescence. Interestingly, we observed the same pattern of results in individuals with obesity [21], suggesting that early experiences of dismissing caring relationships with parents may promote (rather than decrease) the desire and seeking behavior for more satisfying relationships in adulthood, in line with the social reconnection hypothesis [42].

A final consideration regards the results relative to pain. The individual and subjective reports of pain, as measured immediately before the experimental procedure, were significantly linked to the hedonic experience of the imagined touch of the hand: the higher the pain, the less the expected pleasantness associated with a gentle human touch. This evidence may support an interplay between nociception and the processing of the hedonic dimension of touch [1,3,43], but, crucially, it suggests that this relationship may not be mediated by bottom-up somatosensory processes. Our findings may indicate that the experience of pain likely impacts more individual expectations of pleasantness than the actual pleasure: importantly, this observation supports the fundamental role of psychological aspects in the process of pain chronicization in this condition [11]. Pain catastrophizing has been consistently found to be a strong predictor of pain intensity and disability in chronic pain conditions, mainly through overwhelming negative thoughts and rumination about painful experiences [44]. The observed negative association between pain intensity and expectancies of pleasure might be mediated by repetitive, passive, and uncontrollable thoughts about pain. Interestingly, Myga and colleagues [45] showed that the inner reiteration of thoughts about the intensity of tactile stimuli (i.e., weak or strong) altered the tactile perception into the expected direction, even when measured indirectly. Our results might be in support of a “non-intentional”, maladaptive process of self-suggestion about bodily experiences, beyond pain perception. Crucially, the mentioned work points to the possibility of *intentional* manipulations, at least relative to discriminative touch. The possibility to use self-suggestion to target tactile perception and/or painful sensations remains open. Yet, evidence in this direction might have crucial clinical implications for effective pain-related treatments. Moreover, future investigations may introduce implicit neurophysiological measures (for example, skin conductance and heartbeat rate [46]) to implicitly assess the autonomic responses to affective touch in fibromyalgia, allowing the investigation of the effect of self-suggestion on bodily perception while attenuating the influence of the task’s demands (e.g., assessing the effect on actual perception of iterative thinking about pleasantness or pain, without asking for explicit ratings).

This study had some limitations. First, we underlined the absence of an ad hoc control group in this research, even though the inclusion/exclusion criteria were strictly adhered to. Regarding the healthy controls, we used data from previously published studies [21,22], and because of this methodological choice, we observed demographical differences between our two groups. As for the age differences, Cruciani and colleagues reported a comparable preference for slow (i.e., CT-optimal) over fast (i.e., CT-non-optimal) touch across the entire lifespan [47]. We have no reason to expect a significant impact of education on affective touch perception: humans can discern affective from non-affective touch from when they are newborns, suggesting this is a very spontaneous ability. Moreover, all the participants declared to have fully understood the tasks and the associated questions. Another aspect that may be noticed about our participants with fibromyalgia is the higher body mass index compared with our controls. This is not surprising considering that being overweight and clinical obesity are very often associated with pain-related diagnoses, including fibromyalgia [48]; however, obesity seems unrelated to altered perceptions of affective touch [21]. Also, we collected data only from female participants to guarantee a match with the experimenter’s gender, preventing possible confounding effects (e.g., more embarrassment or a higher sexual connotation of touch). Indeed, the perception of affective touch is significantly affected by gender [49]. Moreover, the prevalence of fibromyalgia is larger in females than male and it may be differently experienced by males and females [50,51,52,53,54]. Bearing this in mind, data focused on male participants should be collected. Future investigations may take advantage of a multicentric approach to involve larger and more representative samples to improve the results’ generalizability. Lastly, no information was available about psychosocial wellbeing in healthy women, nor about ongoing pharmacological treatments (even if the inclusion/exclusion criteria may suggest the absence of any ongoing treatment). Painkillers and benzodiazepine may affect the somatosensory sensitivity due to their analgesic and sedative effects; thus, complete knowledge about regularly used drugs, especially regarding the individuals with pain-related disease, may be recommended.

## 5. Conclusions

Our and Boheme and colleagues’ evidence [14] may suggest that women with fibromyalgia can coherently disentangle the level of pleasantness associated with affective versus non-affective touch, even though the experience of pain may affect individual’s expectations about pleasure. Therefore, we encourage future research to verify whether affective touch can be used for restoring purposes, such as reducing pain and increasing body-related health in fibromyalgia as well as to verify the role of top-down components, including individual expectations and internally repeated thoughts [45], on the individual perceptions of bodily signals, including pain and affective touch.

## Figures and Tables

**Table 1:** Mean and standard deviations (SDs) of the TBIO subscales relative to participants with fibromyalgia (*n* = 14) and healthy controls (*n* = 14). Statistics about between-subject comparisons are reported; in bold are the significant differences.

	Participants with Fibromyalgia	Healthy Controls	Statistics
TBIO Subscale			
Childhood/adol.	2.75 (1.02)	3.91 (0.90)	**U = 39, *p* = 0.007, r_b_ = 0.60**
Adulthood	3.63 (0.95)	3.77 (0.84)	U = 94, *p* = 0.87, r_b_ = 0.05
Comfort	4.05 (0.92)	3.77 (0.81)	U = 118, *p* = 0.37, r_b_ = 0.20
Fondness	4.13 (0.71)	4.07 (1.02)	U = 93.50, *p* = 0.85, r_b_ = 0.04

**Table 2:** Correlational results exploring the association between the affective touch sensitivity index and the scores of the TBIO questionnaire, relative to the touch of the hand and the brush in participants with fibromyalgia (*n* = 14) and the healthy controls (*n* = 14) and for both the imagery and the real tasks. Spearman’s rho coefficients and *p*-values (in brackets) are reported.

Tactile Biography Questionnaire					
		Childhood/adolescence	Adult	Comfort	Fondness
**Participants with Fibromyalgia**					
**Imagery**	*Brush*	0.05 (0.43)	0.30 (0.15)	0.37 (0.10)	0.31 (0.14)
	*Hand*	−0.27 (0.82)	−0.13 (0.67)	0.44 (0.06)	−0.01 (0.52)
**Real**	*Brush*	0.21 (0.24)	0.34 (0.12)	0.33 (0.12)	0.41 (0.08)
	*Hand*	0.15 (0.30)	0.34 (0.12)	0.24 (0.21)	0.32 (0.13)
**Healthy Controls**					
**Imagery**	*Brush*	−0.18 (0.74)	0.07 (0.40)	−0.80 (0.61)	−0.24 (0.79)
	*Hand*	0.04 (0.45)	0.02 (0.48)	−0.04 (0.60)	0.025 (0.47)
**Real**	*Brush*	−0.08 (0.60)	−0.001 (0.50)	−0.28 (0.83)	−0.26 (0.82)
	*Hand*	−0.20 (0.75)	0.19 (0.26)	0.004 (0.49)	−0.23 (0.78)

**Table 3:** Correlational results exploring the relationship between the affective touch sensitivity index, relative to both the hand and the brush, and the self-reported level of pain experienced by participants with fibromyalgia (*n* = 14). Spearman’s rho coefficients and *p*-values (in brackets) are reported.

	Pain Intensity	Pain Whole Body ♦	Pain Forearm ♦
**Imagery**			
Brush	−0.16 (0.28)	−0.12 (0.35)	0.25 (0.80)
Hand	−0.35 (0.11)	**−0.55 (0.03)**	**−0.50 (0.04)**
**Real**			
Brush	0.25 (0.81)	−0.05 (0.43)	0.45 (0.94)
Hand	(0.97)	−0.04 (0.44)	0.49 (0.95)

♦ Note: Only 13 participants were included in the correlational analysis between the affective touch sensitivity index and pain since pain ratings for one participant were missing.

## Data Availability

The original data presented in the study are openly available at https://zenodo.org/records/10993443 (accessed on 18 April 2024) (10.5281/zenodo.10993442).

## Supplementary Materials

The following supporting information can be downloaded at https://www.mdpi.com/article/10.3390/jcm13185649/s1: Figure S1: Feelings related to interpersonal touch are represented on the y-axis. Bars illustrate the percentage of participants with fibromyalgia (pink) and healthy participants (light blue) reporting certain feelings; * significant differences according to the χ^2^ statistic. Table S1: Percentages (n) of participants with fibromyalgia and healthy women who experienced negative interpersonal bodily contact, give their preference either for giving and/or receiving affective touch, and associate certain feelings in interpersonal bodily contacts. Statistics relative to the chi-squared tests are reported included χ^2^, *p* values and Cramer’s V: significant associations are in bold.

## References

[1] Meijer L.L., Ruis C., van der Smagt M.J., Scherder E.J.A., Dijkerman H.C. (2022). Neural Basis of Affective Touch and Pain: A Novel Model Suggests Possible Targets for Pain Amelioration. J. Neuropsychol..

[2] Vallbo Å.B., Olausson H., Wessberg J. (1999). Unmyelinated Afferents Constitute a Second System Coding Tactile Stimuli of the Human Hairy Skin. J. Neurophysiol..

[3] Choi S., Hachisuka J., Brett M.A., Magee A.R., Omori Y., Iqbal N.-A., Zhang D., DeLisle M.M., Wolfson R.L., Bai L. (2020). Parallel Ascending Spinal Pathways for Affective Touch and Pain. Nature.

[4] Krahé C., Drabek M.M., Paloyelis Y., Fotopoulou A. (2016). Affective Touch and Attachment Style Modulate Pain: A Laser-Evoked Potentials Study. Philos. Trans. R. Soc. B Biol. Sci..

[5] Di Lernia D., Lacerenza M., Ainley V., Riva G. (2020). Altered interoceptive perception and the effects of interoceptive analgesia in musculoskeletal, primary, and neuropathic chronic pain conditions. J. Pers. Med..

[6] Habig K., Lautenschläger G., Maxeiner H., Birklein F., Krämer H.H., Seddigh S. (2021). Low Mechano-Afferent Fibers Reduce Thermal Pain but Not Pain Intensity in CRPS. BMC Neurol..

[7] Fusaro M., Bufacchi R.J., Nicolardi V., Provenzano L. (2022). The Analgesic Power of Pleasant Touch in Individuals with Chronic Pain: Recent Findings and New Insights. Front. Integr. Neurosci..

[8] Ashburn M.A., Staats P.S. (1999). Management of Chronic Pain. Lancet.

[9] Wolfe F., Clauw D.J., Fitzcharles M.-A., Goldenberg D.L., Häuser W., Katz R.L., Mease P.J., Russell A.S., Russell I.J., Walitt B. (2016). 2016 Revisions to the 2010/2011 Fibromyalgia Diagnostic Criteria. Semin. Arthritis Rheum..

[10] Kosek E., Cohen M., Baron R., Gebhart G.F., Mico J.-A., Rice A.S.C., Rief W., Sluka A.K. (2016). Do We Need a Third Mechanistic Descriptor for Chronic Pain States?. Pain.

[11] Bhargava J., Hurley J.A. (2024). Fibromyalgia.

[12] Yuan S.L.K., Matsutani L.A., Marques A.P. (2015). Effectiveness of Different Styles of Massage Therapy in Fibromyalgia: A Systematic Review and Meta-Analysis. Man. Ther..

[13] Li Y., Wang F., Feng C., Yang X., Sun Y. (2014). Massage Therapy for Fibromyalgia: A Systematic Review and Meta-Analysis of Randomized Controlled Trials. PLoS ONE.

[14] Boehme R., Van Ettinger-Veenstra H., Olausson H., Gerdle B., Nagi S.S. (2020). Anhedonia to Gentle Touch in Fibromyalgia: Normal Sensory Processing but Abnormal Evaluation. Brain Sci..

[15] Nanay B. (2018). Multimodal mental imagery. Cortex.

[16] Pearson J., Kosslyn S.M. (2015). The heterogeneity of mental representation: Ending the imagery debate. Proc. Natl. Acad. Sci. USA.

[17] Lucas M.V., Anderson L.C., Bolling D.Z., Pelphrey K.A., Kaiser M.D. (2015). Dissociating the neural correlates of experiencing and imagining affective touch. Cereb. Cortex.

[18] McGlone F., Wessberg J., Olausson H. (2014). Discriminative and Affective Touch: Sensing and Feeling. Neuron.

[19] Nordin M. (1990). Low-threshold mechanoreceptive and nociceptive units with unmyelinated (C) fibres in the human supraorbital nerve. J. Physiol..

[20] Augière T., Desjardins A., Paquette Raynard E., Brun C., Pinard A.M., Simoneau M., Mercier C. (2021). Tactile Detection in Fibromyalgia: A Systematic Review and a Meta-Analysis. Front. Pain Res..

[21] Tagini S., Scacchi M., Mauro A., Scarpina F. (2023). The Perception of Affective Touch in Women Affected by Obesity. Front. Psychol..

[22] Tagini S., Bastoni I., Villa V., Mendolicchio L., Castelnuovo G., Mauro A., Scarpina F. (2023). Affective Touch in Anorexia Nervosa: Exploring the Role of Social Anhedonia and Lifespan Experiences. J. Affect. Disord..

[23] Beltrán M.I., Dijkerman H.C., Keizer A. (2020). Affective Touch Experiences across the Lifespan: Development of the Tactile Biography Questionnaire and the Mediating Role of Attachment Style. PLoS ONE.

[24] Sailer U., Ackerley R. (2019). Exposure Shapes the Perception of Affective Touch. Dev. Cogn. Neurosci..

[25] Wolfe F., Hawley D.J., Wolfe F., Hawley D.J. (1998). Psychosocial Factors and the Fibromyalgia Syndrome. Z. Rheumatol..

[26] Anderberg U.M., Marteinsdottir I., Theorell T., von Knorring L. (2000). The Impact of Life Events in Female Patients with Fibromyalgia and in Female Healthy Controls. Eur. Psychiatry.

[27] Wolfe F., Walitt B.T., Katz R.S., Häuser W. (2014). Symptoms, the Nature of Fibromyalgia, and Diagnostic and Statistical Manual 5 (DSM-5) Defined Mental Illness in Patients with Rheumatoid Arthritis and Fibromyalgia. PLoS ONE.

[28] D’Onghia M., Ciaffi J., Lisi L., Mancarella L., Ricci S., Stefanelli N., Meliconi R., Ursini F. (2021). Fibromyalgia and Obesity: A Comprehensive Systematic Review and Meta-Analysis. Semin. Arthritis Rheum..

[29] Langley G.B., Sheppeard H. (1985). The Visual Analogue Scale: Its Use in Pain Measurement. Rheumatol. Int..

[30] Grossi E., Mosconi P., Groth N., Niero M., Apollone G. (2002). Questionario Psychological General. Well-Being Index: Versione Italiana.

[31] Monticone M., Giorgi I., Baiardi P., Barbieri M., Rocca B., Bonezzi C. (2010). Development of the Italian Version of the Tampa Scale of Kinesiophobia (TSK-I): Cross-Cultural Adaptation, Factor Analysis, Reliability, and Validity. Spine.

[32] Monticone M., Baiardi P., Ferrari S., Foti C., Mugnai R., Pillastrini P., Rocca B., Vanti C. (2012). Development of the Italian Version of the Pain Catastrophising Scale (PCS-I): Cross-Cultural Adaptation, Factor Analysis, Reliability, Validity and Sensitivity to Change. Qual. Life Res..

[33] Crucianelli L., Cardi V., Treasure J., Jenkinson P.M., Fotopoulou A. (2016). The perception of affective touch in anorexia nervosa. Psychiatry Res..

[34] Olausson H., Wessberg J., Morrison I., McGlone F. (2016). Affective Touch and the Neurophysiology of CT Aff Erents.

[35] Löken L.S., Wessberg J., Morrison I., McGlone F., Olausson H. (2009). Coding of Pleasant Touch by Unmyelinated Afferents in Humans. Nat. Neurosci..

[36] Ojala J., Suvilehto J.T., Nummenmaa L., Kalso E. (2023). Bodily Maps of Emotions and Pain: Tactile and Hedonic Sensitivity in Healthy Controls and Patients Experiencing Chronic Pain. Pain.

[37] Case L.K., Čeko M., Gracely J.L., Richards E.A., Olausson H., Bushnell M.C. (2016). Touch Perception Altered by Chronic Pain and by Opioid Blockade. eNeuro.

[38] Faul F., Erdfelder E., Lang A.G., Buchner A. (2007). G*Power 3: A flexible statistical power analysis program for the social, behavioral, and biomedical sciences. Behav. Res. Methods.

[39] Romeo A., Di Tella M., Ghiggia A., Tesio V., Fusaro E., Geminiani G.C., Castelli L. (2020). Attachment Style and Parental Bonding: Relationships with Fibromyalgia and Alexithymia. PLoS ONE.

[40] Imbierowicz K., Egle U.T. (2003). Childhood Adversities in Patients with Fibromyalgia and Somatoform Pain Disorder. Eur. J. Pain.

[41] Gil F.P., Weigl M., Wessels T., Irnich D., Baumüller E., Winkelmann A. (2008). Parental Bonding and Alexithymia in Adults with Fibromyalgia. Psychosomatics.

[42] Maner J.K., DeWall C.N., Baumeister R.F., Schaller M. (2007). Does Social Exclusion Motivate Interpersonal Reconnection? Resolving the “Porcupine Problem”. J. Pers. Soc. Psychol..

[43] Chen L.M. (2018). Cortical Representation of Pain and Touch: Evidence from Combined Functional Neuroimaging and Electrophysiology in Non-Human Primates. Neurosci. Bull..

[44] Simic K., Savic B., Knezevic N.N. (2024). Pain Catastrophizing: How Far Have We Come. Neurol. Int..

[45] Myga K.A., Kuehn E., Azañón E. (2024). How the inner repetition of a desired perception changes actual tactile perception. Sci. Rep..

[46] Mazza A., Cariola M., Capiotto F., Diano M., Schintu S., Pia L., Dal Monte O. (2023). Hedonic and Autonomic Responses in Promoting Affective Touch. Sci. Rep..

[47] Cruciani G., Zanini L., Russo V., Mirabella M., Palamoutsi E.M., Spitoni G.F. (2021). Strengths and weaknesses of affective touch studies over the lifetime: A systematic review. Neurosci. Biobehav. Rev..

[48] Vaioli G., Scarpina F. (2021). Facial Emotion Recognition in Obesity and in Fibromyalgia: A Systematic Review. NeuroSci.

[49] Russo V., Ottaviani C., Spitoni G.F. (2020). Affective touch: A meta-analysis on sex differences. Neurosci. Biobehav. Rev..

[50] Buskila D., Neumann L., Alhoashle A., Abu-Shakra M. (2000). Fibromyalgia syndrome in men. Semin. Arthr. Rheum..

[51] Rodham K., Rance N., Blake D. (2010). A qualitative exploration of carers’ and ‘patients’ experiences of fibromyalgia: One illness, different perspectives. Musculoskelet. Care.

[52] Yunus M.B., Celiker R., Aldag J.C. (2004). Fibromyalgia in men: Comparison of psychological features with women. J. Rheumatol..

[53] Cohen H., Neumann L., Alhosshle A., Kotler M., Abu-Shakra M., Buskila D. (2001). Abnormal sympathovagal balance in men with fibromyalgia. J. Rheumatol..

[54] Miró E., Martínez M.P., Sánchez A.I., Prados G., Lupiáñez J. (2015). Men and women with fibromyalgia: Relation between attentional function and clinical symptoms. Br. J. Health. Psychol..

