# Supplementary material for: The Hedonic Experience Associated with a Gentle Touch Is Preserved in Women with Fibromyalgia
Source: J Clin Med. 2024 Sep 23;13(18):5649. doi: 10.3390/jcm13185649 (PMC11433436; doi:10.3390/jcm13185649)
Supplement: Supplementary file 1 [file jcm-13-05649-s001.zip › jcm-3152995-supplementary.pdf]

The hedonic experience associated with a gentle touch is preserved in women with fibromyalgia

Supplementary Materials

Sofia Tagini, Giorgia Varallo, Paolo Capodaglio, Alessandro Mauro, & Federica Scarpina

S1 TBIO supplementary items

In line with the original validation of the instrument (Beltrán et al., 2020) and a recent work exploring the use of this questionnaire in a sample of Italian people (Mariani Wigley et al., 2022), we included in formal analyses only the first 29 items of the questionnaire, which were all scored from 1 to 5. For clarity sake, we reported TBIO extra information about the i) *feelings* experienced in bodily affective interactions, ii) previous *negative/unpleasant experiences* involving interpersonal touch and iii) the participant's *preference* for giving and/or receiving affective touch as supplement to our work.

The chi-square test was used to investigated whether the *Group* (participants with fibromyalgia *vs* healthy controls) was significantly associated with the presence of negative experiences of interpersonal touch, the preference for giving and/or receiving affective touch, and the occurrence of certain pleasant or unpleasant feelings in bodily affective interactions. Two-sided, exact, p values ≤ 0.05 were considered statistically significant.

Table S1. Percentages (*n*) of participants with fibromyalgia and healthy women who experienced negative interpersonal bodily contact, give their preference either for giving and/or receiving affective touch, and associate certain feelings in interpersonal bodily contacts. Statistics relative to the chi-squared tests are reported included χ2, p values and Cramer's V: significant associations are in bold.

	**Participants with fibromyalgia**	**Healthy controls**	**χ^2 Statistics**
**Preference for giving and/or receiving affective touch**			
Give affective touch	28.57% (*n*=4)	14.29% (*n*=2)	
Receive affective touch	7.14 % (*n*=1)	35.71 % (*n*=5)	χ^2 =3.62, *p*= 0.31, *V*=0.36
Both give and receive	57.14% (*n*=8)	42.86 % (*n*=6)	
Neither give or receive	7.14% (*n=1*)	7.14% (*n*=1)	
**Unpleasant experience of interpersonal touch in life**	84.62 % (*n*=11) ♦	23.10% (*n*=4)	**χ^2=6-09, *p*=0.003, *V*=0.56**
**Feelings associated with interpersonal touch**			
Relax	50% (*n*=7)	92.31% (*n*=13)	**χ^2=6.30, *p*=0.01, *V*=0.47**
Pleasure	35.71% (*n*=5)	0 % (n=0)	**χ^2=6.09, *p*=0.01, *V*=0.47**
Embarrassment	7.14% (1)	30.77% (4)	χ^2=2.49, *p*=0.11, *V*=0.30
Satisfaction	42.86% (6)	61.54% (8)	χ^2=0.94, *p*=0.33, *V*=0.19
Happiness	42.86% (6)	61.54% (8)	χ^2=0.94, *p*=0.33, *V*=0.19
Rejection	7.14% (1)	0%	χ^2=0.96, *p*=0.33, *V*=0.19
Disgust	0%	0%	-
Comfort	71.43% (10)	92.31% (12)	χ^2=1.95, *p*=0.16, *V*=0.27
Annoyance	7.14% (1)	0%	χ^2=0.96, *p*=0.33, *V*=0.19
Uneasiness	7.14% (1)	0%	χ^2=0.96, *p*=0.33, *V*=0.19

♦ *Percentage is computed on 13 participants with fibromyalgia due to missing information.*

These additional observations suggest that overall our participants mostly like both giving and receiving affective touch. Relative to the healthy controls, the remaining participants clearly preferred to receive, whereas women with fibromyalgia would rather give affective touch. However, there was no significant association between the expressed preference and the group (*p*= 0.31). Importantly,

participants with fibromyalgia reported more frequently negative / unpleasant experiences involving interpersonal touch than healthy controls. Accordingly, as illustrated in Figure S1, relaxing and healing sensations were less frequently associated with affective touch by women with fibromyalgia than healthy controls. However, and surprisingly, one into three participants with fibromyalgia associated pleasure with interpersonal touch, whereas none of the healthy controls did so. This observation among healthy controls is certainly unexpected. Moreover, previous works investigating the validity of the TBIO questionnaire (Beltrán et al., 2020; Mariani Wigley et al., 2022) did not report information about nor discuss experimental findings relative to this specific item, preventing from any sound interpretation of this oddness. From a speculative perspective, we suggest that the term "pleasure" may evoke a specific, erotic, context of touch, which is clearly restricted to singular relationships; in fact, participants were asked to think about interpersonal contact in a broader sense. The reason why – a few minority of - participants with fibromyalgia did not so remains an open question. On the other hand, in both groups, most of participants reported satisfaction, happiness, comfort, and embarrassment relative to affective touch; negative feelings (i.e., rejection, annoyance, and disgust) were rarely reported in association with affective touch in both groups.

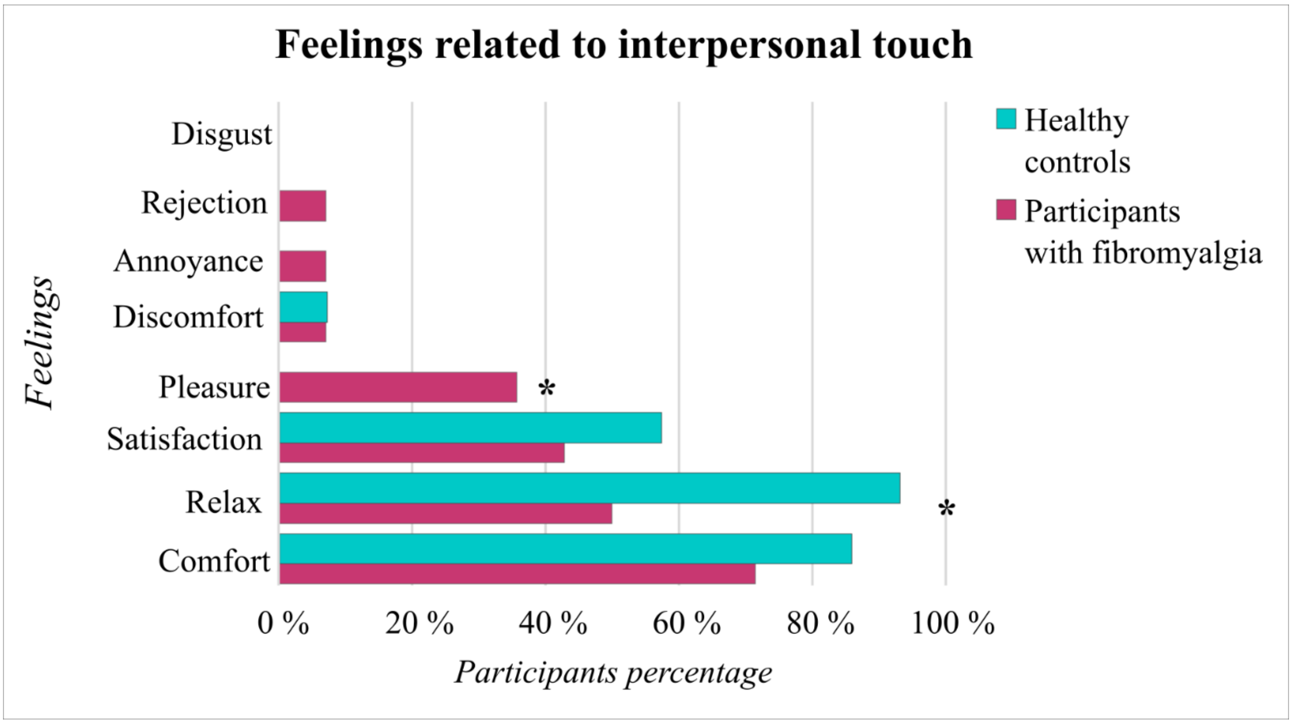

Figure S1. Feelings related to interpersonal touch are represented on the y-axis. Bars illustrate the percentage of participants with fibromyalgia (pink) and healthy participants (light blue) reporting certain feelings; * significant differences according to the χ^2 statistic.
